# Dynamics of tactical behaviour in association football when manipulating players' space of interaction

**DOI:** 10.1371/journal.pone.0180773

**Published:** 2017-07-14

**Authors:** Angel Ric, Carlota Torrents, Bruno Gonçalves, Lorena Torres-Ronda, Jaime Sampaio, Robert Hristovski

**Affiliations:** 1 Complex Systems in Sport Research Group, National Institute of Physical Education of Catalonia (INEFC), University of Lleida, Lleida, Spain; 2 Research Center in Sports Sciences, Health Sciences and Human Development (CIDESD), CreativeLab Research Community, Universidade de Trás-os-Montes e Alto Douro, Vila Real, Portugal; 3 Department of Health and Kinesiology Exercise and Sport Nutrition Lab, Texas A&M University, College Station, Texas, United States of America; 4 Faculty of Physical Education, Sport and Health, Saint Cyril and Methodious University, Skopje, Macedonia; Nanyang Technological University, SINGAPORE

## Abstract

The analysis of positional data in association football allows the spatial distribution of players during matches to be described in order to improve the understanding of tactical-related constraints on the behavioural dynamics of players. The aim of this study was to identify how players’ spatial restrictions affected the exploratory tactical behaviour and constrained the perceptual-motor workspace of players in possession of the ball, as well as inter-player passing interactions. Nineteen professional outfield male players were divided into two teams of 10 and 9 players, respectively. The game was played under three spatial constraints: a) players were not allowed to move out of their allocated zones, except for the player in possession of the ball; b) players were allowed to move to an adjacent zone, and; c) non-specific spatial constraints. Positional data was captured using a 5 Hz interpolated GPS tracking system and used to define the configuration states of players for each second in time. The configuration state comprised 37 categories derived from tactical actions, distance from the nearest opponent, distance from the target and movement speed. Notational analysis of players in possession of the ball allowed the mean time of ball possession and the probabilities of passing the ball between players to be calculated. The results revealed that the players’ long-term exploratory behaviour decreased and their short-term exploration increased when restricting their space of interaction. Relaxing players’ positional constraints seemed to increase the speed of ball flow dynamics. Allowing players to move to an adjacent sub-area increased the probabilities of interaction with the full-back during play build-up. The instability of the coordinative state defined by being free from opponents when players had the ball possession was an invariant feature under all three task constraints. By allowing players to move to adjacent sub-areas, the coordinative state became highly unstable when the distance from the target decreased. Ball location relative to the scoring zone and interpersonal distance constitute key environmental information that constrains the players’ coordinative behaviour. Based on our results, dynamic overlap is presented as a good option to capture tactical performance. Moreover, the selected collective (i.e. relational) variables would allow coaches to identify the effects of training drills on teams and players’ behaviour. More research is needed considering these type variables to understand how the manipulation of constraints induce a more stable or flexible dynamical structure of tactical behaviour.

## Introduction

The analysis of performance in sport through the collection and subsequent processing of data has been widely used to provide useful information for coaches [1]. This performance analysis has sought to obtain indicators of execution, such as offensive technical actions or successful defensive events, and has ranked them using statistical procedures to characterize football performance [2–5]. Although these previous studies have led to advances in football performance, the notation of discrete actions and/or events has not provided information about the certain performance contexts [6]. For instance, ball location relative to the scoring targets constrains the emergence of spatiotemporal coordinated team behaviours [7]. Various studies have employed other measures such as players’ trajectories, interpersonal distances, relative angles between players or velocities as state variables [8–11]. They have served to define the coordination system states (patterns) at different levels of analysis (i.e., player, team or game). Hence, the challenge in performance analysis is to capture key contextual information that helps to describe and model the varied game scenarios explored by the team and players during both training and competition [12,13].

A combination of ball events and positional data is needed to understand the players’ and team’s performance. Thus, several indicators such as player-player and player-ball dyadic coordination, intra- and inter-team synchronization, pattern-forming dynamics, time required to regain ball possession, ball possession percentage, number of passes and their length have been used to characterize individual and collective performance [14–17]. The notation of passes has also helped to quantify the main interactions between the player in possession of the ball and her or his teammates. These data can be used to measure closeness and betweenness centrality scores [18–20], flow motifs [21] or a combination of zone and player passing measures [22] to identify team and player performance and styles of play. Accordingly, Yamamoto and Yokoyama [23] found the existence of a power law structure in the network of passing behaviour. This degree distribution suggests that self-organisation phenomena occurs during ball flow football dynamics and characterize a scale-free network where few players acts as a game hub. Due to the small number of nodes (11 players), Narizuka and colleagues [24] included the player location when performing a pass. They found that the degree distributions fitted with a truncated gamma distribution, showing that each player moves around his own home position when interacting with the ball. Cotta and colleagues [25] also identified this positional feature when characterizing the Spanish national team style of play, by analysing passing network during the 2010 FIFA world cup. Such ball-passing dynamics not only influence the teams’ dynamics, but also the local players’ dynamics (e.g., movement reconfigurations) [26,27]. That is, after a pass, the player in possession of the ball switches, change the topology of the passing network (i.e. ball-passing probability distribution) and the perceptual-motor workspace of the player in possession of the ball.

Football players’ perceptual-motor workspace is the dynamic interface between information flows coming from perception, and kinetic flows from action [28,29]. The channelling of players’ exploratory activity is provided by affordances, i.e., possibilities for action mainly offered by the environment [30,31]. In fact, they map environmental information onto movement behaviour within the perceptual-motor workspace in accordance with the demands of the task goal. Due to the multiple ways to score a point in the football game, there is no predetermined interpersonal coordination to be executed in order to attain the goal. Hence, there is a lack of convergence of the exploratory behaviour towards a pre-defined intra- or interpersonal configuration. This situation allows very subtle and consequently unpredictable interactions between the environmental information flow and the performer’s organismic constraints, to decide which particular action, from the perceptual-motor workspace, will be performed at each moment.

These informational variables constrain the system, pushing it towards different states of organization or concrete coordination patterns [32,33]. The manipulation of constraints, especially those related to the tasks, will induce the stability of patterns and the functional exploration through the transitions between organizational states inherent to sports teams [34]. The adequate modification or variations of this type of constraints can produce functional changes in behaviour in competitive and performance contexts. These qualitative changes in the coordinative system states are characterized by the loss of stability from a previous state to the current one. In this sense, behavioural dynamics correspond to the change of trajectories, i.e. bifurcations, that occur in the performance state space, making reference to the whole set of possible coordinative states of a system [35].

Key environmental information, and the manipulation of task constraints, will allow players to explore a variety of functional movements. That is to say, in order to foster the emergence of functional tactical behaviours, coaches should manipulate training drills enhancing the number of possibilities for action that can satisfy goals, or suppressing habitual behaviours [36,37]. In individual sports, it has been experimentally demonstrated that constraining such habitual behavioural modes can promote the emergence of novel behaviours. In fact, this manipulation promotes an indirect release of constraints and increases the likelihood of specific emergent behaviour and/or enhances the potential for exploratory behaviour [36,38]. This exploration enhancing mechanism was called ‘the connected door effect’, and it enables greater fluency and flexibility as well as innovative actions [39].

In association football, several studies have reported the effects of manipulating task constraints. For example, increasing the number of goals amplifies possibilities for scoring and, consequently, a stabilization of protecting goal patterns will likely emerge [40]. The higher the number of players in small-sided games requires a higher level of collective organization and optimized space occupation [41]. The number of players involved in the match clearly affects the stability of the team structure, constrains players to using a specific pitch location and defines roles during the match. Curiously, when increasing the relative space per player by reducing the number of players, the players’ positioning tends to be more irregular; however, when the field dimension increases that tendency changes and players’ positioning becomes more regular [42].

Furthermore, Vilar and colleagues [43] found that, for most of the match time, two team members were located in center-back and middle sub-areas of the effective playing space while one player was located in the back- and front-wing and center-front sub-areas. However, their results showed that players’ numerical relations were differently distributed depending on the sub-areas, postulating that local numerical dominance plays a key role in offensive and defensive success. Several studies have explored the influence of numerical imbalances on performance [44–46]. For example, Gonçalves and colleagues [47] analyzed the effects on tactical behaviour when playing with different numbers of opponents or teammates and found higher values of irregularity and variability in players’ positioning relative to their centroid (i.e., the geometrical center of the team) when playing with more than one teammate or opponent. Moreover, under numerical superiority, the players’ distance from their nearest opponent increased, expanding the space and time for interaction. Conversely, Ric and colleagues [48] analyzed the similarity of individual tactical patterns and found that numerical superiority allowed players to explore more varied configurations. However, it was reported that this capacity to explore different patterns expired in time lags up to 20-to-30 seconds, recommending the use of wild-card players that enter and leave the game in that timescale. In basketball, Bourbousson and colleagues [49] found that disruption in inter-team coordination fosters the drive action, proposing a task including an extra player, who systematically plays in offense to perturb the coordination modes of defenders. These situations require an increase in players’ breadth of attention to solve the numerical disadvantage, allowing the player in possession of the ball and the supporting players to act within an advantageous context of play. These findings may encourage coaches to use task constraints to preserve the players’ specific locations and roles, as well as the local and temporary nature of numerical imbalances in order to respect the representative nature of training drills.

According to all the presented literature, a task was designed where players were constrained to create local imbalances while preserving their specific playing location and roles. The effects of these constraints on the exploratory dynamics and behavioural tendencies remain unknown. Therefore, the aim of this study was to identify how players’ spatial restrictions affected their exploration of tactical behaviour and constrained the perceptual-motor workspace of players in possession of the ball. We hypothesized that spatial restrictions would increase the rate and breath of player’s exploratory behaviour. Moreover, coach staff expected that these task constraints would allow players to possess the ball far from the opponent in closer zones to the target, enhancing the passing flow.

## Methods

### Participants and procedures

**Fig 1:**
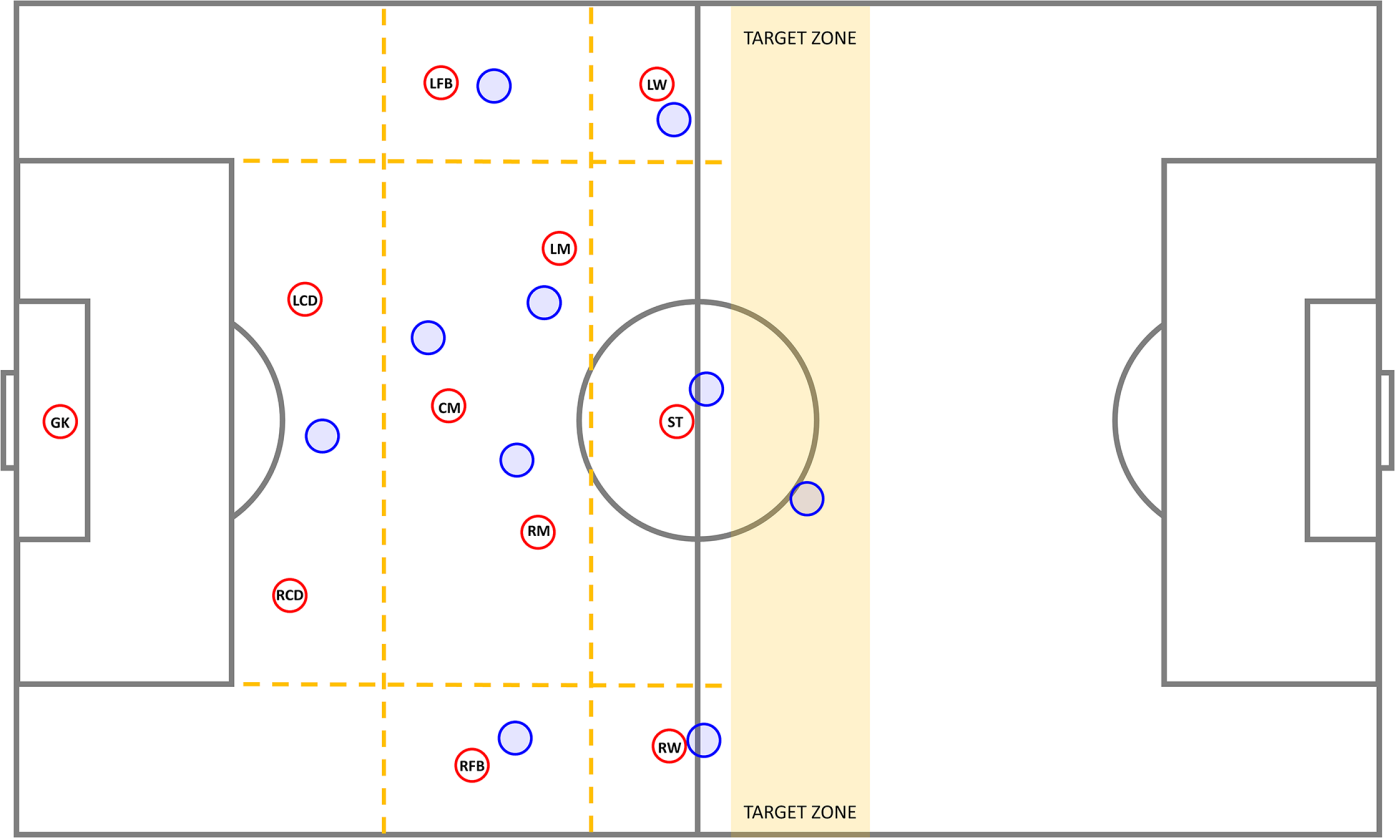
Schematic representation of the experimental task.

Twenty-one male professional football players (age: 25.1±4.1 years; playing experience: 18.8±5.3 years) participated in the study. All the players were informed about the procedures and signed a consent form to participate voluntarily in the study. Players were divided into two teams, taking into account their playing positions and their physical, technical and tactical levels according to a coach’s subjective criteria. This was done to ensure that the performance level of each team to be comparable [50]. The analyzed team comprised ten outfield players plus a goalkeeper. The opposing team comprised nine outfield players and a goalkeeper. The opponent goalkeeper was placed in the opponent target zone situated 37.5 m away from the penalty area, supporting the offensive action of his teammates as an extra player. Based on the expert knowledge of experienced football coaches and on earlier studies [43,51], the pitch was divided into nine different sub-areas between the penalty area and the target zone, and the players were placed according to their position roles (see Fig 1). The head coach then constrained the players’ movements between the sub-areas according to the following instructions: a) players were not allowed to move out of the sub-areas during the game, except for the player in possession of the ball; b) all players were allowed to move to any adjacent sub-area, and; c) non-specific spatial constraints. Each of these situations was played for a 5-minute period with a 3-minute period of passive rest. The three situations were repeated twice in a randomized order. The sample size was determined by a power analysis, computed using G*Power 3.1 [42] for an effect size of d = 1, α < 0.05, power (1 – β) = 0.95. In similar studies of exploratory dynamics [38], large effect sizes were observed (the smallest value was d = 0.84). The study was approved by the Clinical Research Ethics Committee of the Catalan Institute of Health, University Hospital Arnau de Vilanova, Generalitat de Catalunya (Project-CEIC 1325), which follows the recommendations of the Declaration of Helsinki.

### Data collection

All players wore, on their upper backs, a GPS device (SPI-Pro, GPSports, Canberra, ACT, Australia) that captured the latitudinal and longitudinal coordinates with a sampling frequency of 5 Hz. The time series were exported and transformed into a Cartesian coordinate system using dedicated routines in Matlab R2014b (MathWorks, Inc., Massachusetts, USA) (see Folgado et al. [15] for complete guidelines). The players’ positioning data were resampled using two-frames moving average filter to reduce tracking error noise. In addition, the player who received the ball was notated, as was the time that elapsed between his reception of the ball and that of another teammate before a pass. The dynamic players’ positional data was used to calculate movement speed [15], distance from the nearest opponent [52], distance from the target [53] and tactical actions [48,54]. Tactical actions were estimated from players’ trajectories relative to the position of the player in possession of the ball. The data collected for each player yielded configuration states derived from the 37 variables pertaining to the four tactical measures named above (see Table 1). Players changed their states during the 5-minute game (i.e., 300 seconds each game). Thus, every window of one second was defined as a 37-component binary vector (column) representing the full configuration state, ascribing a value of 1 for active categories and 0 for the inactive ones. This enabled the formation of a temporal 37 x 300 multivariate binary (Boolean) matrix.

**Table 1:** Data collected to assess the tactical patterns of each player, formed by 37 categories. Each data vector represented a player’s configuration in a 4D-category space.

VARIABLE (number of categories per variable)	Category number	CATEGORIES (37)
Tactical actions (10)	1	Penetration
	2	Offensive coverage
	3	Depth mobility
	4	Width and length
	5	Offensive unity
	6	Delay
	7	Defensive coverage
	8	Balance
	9	Concentration
	10	Defensive unity
Distance from the target (9)	11	>37.45 m.
	12	32.1–37.45 m.
	13	36.75–32.1 m.
	14	21,4–26.75 m.
	15	16.05–21.4 m.
	16	10.7–16.05 m.
	17	5.35–10.7 m.
	18	0–5.35 m.
	19	<0 m.
Distance from the nearest opponent (12)	20	<1 m.
	21	1–2 m.
	22	2–3 m.
	23	3–4 m.
	24	4–5 m.
	25	5–6 m.
	26	6–7 m.
	27	7–8 m.
	28	8–9 m.
	29	9–10 m.
	30	10–11 m.
	31	>11 m.
Movement speed (6)	32	<0.7 km · h−1 (stand)
	33	0.7–3.6 km · h−1 (walk)
	34	3.6–7.2 km · h−1 (jog)
	35	7.2–14.4 km · h−1 (medium-intensity running)
	36	14.4–19.8 km · h−1 (high-intensity running)
	37	>19.8 km · h−1 (sprint)

### Data analysis

Dynamic overlap q_*d*_(*t*) was used to determine the region of the performer-environment state space explored by the players and the rate of exploration on different timescales [38,48,55]. The overlap was defined as a cosine similarity between two binary configuration vectors at ever-increasing time distances (i.e., time lags), capturing the mean similarity of configuration states. The mean dynamic overlap was then fitted by the following equation, which is derived for systems with an intricate hierarchical structure (see Sibani & Dall [56]):

$$<q_{d}(t)>=(1-q_{stat})t^{\alpha }+q_{stat}$$

**Fig 2:**
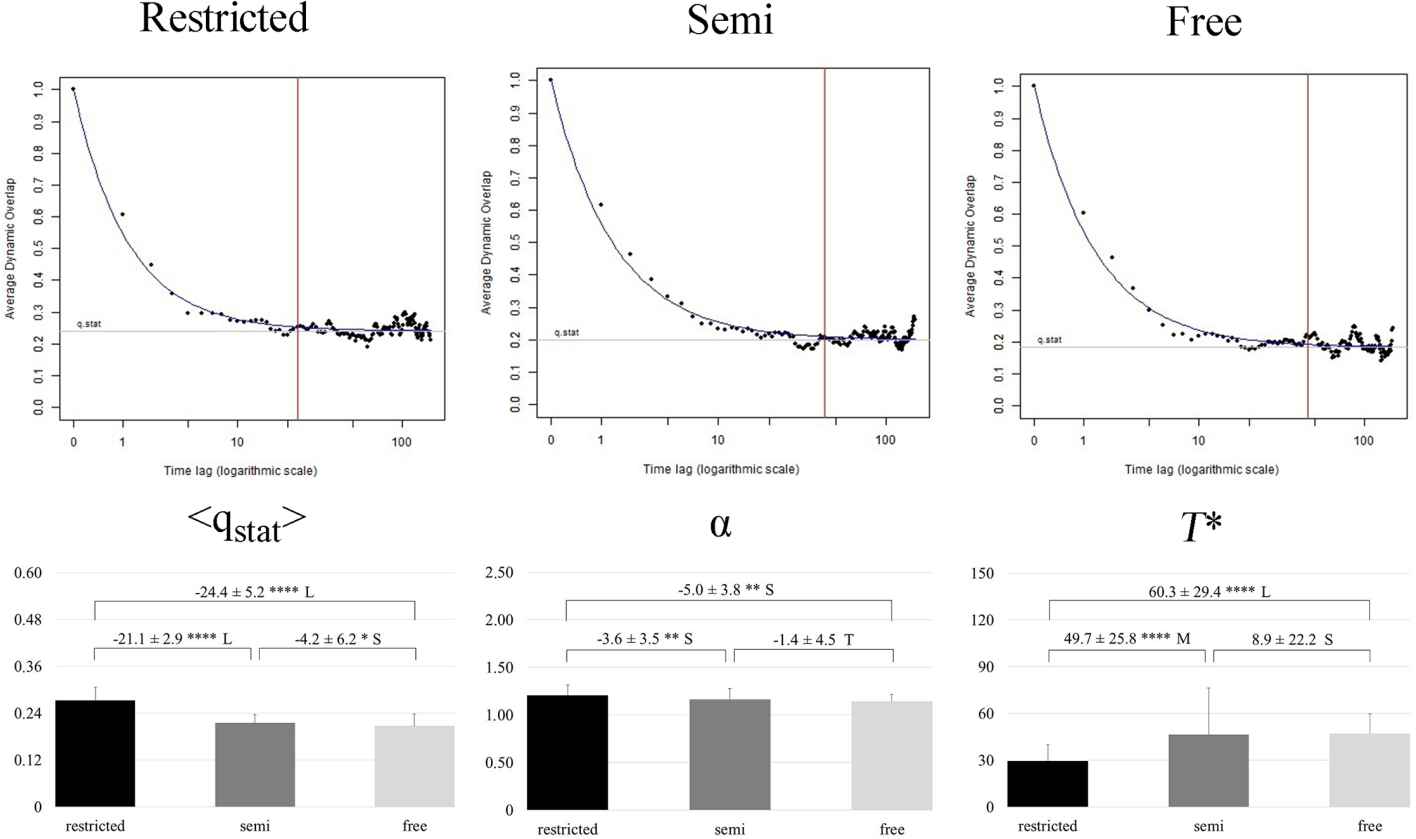
The upper panels show an example of evolution in the mean dynamic overlap of the same player for three different task constraints of a player: *restricted* (left), *semi* (center), *free* (right). The blue lines represent the adjusted curve to the non-linear function, the grey lines represent the stationary <q_stat_> value when the curve tends to infinity, the red lines represent the time lag in which, for a fixed value of 0.05, the asymptotic value intersects with the curve. The lower panels show the mean values for <q_stat_> (left), α exponent (center) and time lag (right). Differences in mean are expressed as percentages (±90% CL). The asterisks indicate the likelihood for the magnitude of the true difference in means as follows: *possible; **likely; ***very likely; ****most likely. The letters denote the effect sizes: T = trivial; S = small; M = moderate; L = large.

From the known mean dynamic overlap (<*q*_*d*_*(t)>)* at every time lag (*t*), three different parameters were calculated. *q*_*stat*_ is the asymptotic value of the dynamic overlap (i.e. the horizontal line towards which the curve, adjusted by the equation, tends to infinity). The α parameter is the slope of the curve. Finally, T* is the time point where, for a fixed value of 0.05, the asymptotic value intersects with the curve of the non-linear model (see upper panels of Fig 2 for a better interpretation).

In order to understand the ball dynamics, the pooled means of time that a player was in possession of the ball before passing it to a teammate, losing it or scoring a goal were calculated. The total passes performed with successful reception by a teammate, as well as turnovers (losing possession of the ball) and goals scored were also taken into account, obtaining the relative frequencies of players’ passing interactions. Transition probabilities were calculated dividing the number of each player’s passes to his teammates, turnovers and goals by the total number of player interactions. Thus, the most probable sequences of passes between players allowed the inter-player interaction tendencies and the characteristic passing channels to reach the target/scoring zone to be identified.

In addition, distance from the nearest opponent and distance from the target were the state variables, and the 2D space that they spanned was the configuration (state) space of the player in possession of the ball. The configuration space contains all possible configurations under the task constraints. The relative frequencies of the player in possession of the ball’s configuration space were taken into account for each task constraint. The probabilities of each configuration were calculated as limit (large N) relative frequencies for stationary distributions: pi=niN, where n_i_ is the frequency of the configuration and N is the total frequencies. This data allowed a potential landscape to be depicted, which represented the configuration space of the player in possession of the ball for each task constraint. The potential values V_i_ were calculated following [57] as: Vi=Qln(PiN); where Q = 1 is the standardized variance term; and N is the total number of configurations in the space spanned by the two state variables.

### Statistical analysis

Practical differences among task constraints were analysed using magnitude-based inferences [58]. All data were first log-transformed to reduce bias arising from non-uniformity error. A descriptive analysis was performed using mean and standard deviations for each variable (the mean shown is the back-transformed mean of the log transformation). Uncertainty in the differences was expressed as 90% of confidence limits (CL). Quantitative chances were assessed qualitatively and reported using the following scale: 25−75%, possible; 75−95%, likely; 95−99%, very likely; >99%, most likely. A difference was assessed as being unclear if the confidence interval (CI) overlapped both substantially positive and negative thresholds. Cohen’s *d* effect size at 90% CL was calculated using pooled standard deviation for comparisons, and the magnitude ranges for mean differences were: 0–0.2 trivial; >0.2–0.6 small; >0.6–1.2 moderate; >1.2–2 large; >2 very large [48]. In addition, the Chi-square test was used to compare the frequencies of players’ interactions and frequencies of the configuration space between the three task constraints.

## Results

### Dynamics of players’ exploratory behaviour

Dynamic overlap analysis allowed the slow dynamics on a long timescale (where players’ exploration became sufficiently saturated), and the quick dynamics on a shorter timescale (related to the initial relaxation part of the overlap) to be determined (see upper panels of Fig 2 as an example of one player for each task constraint). The lower panel of Fig 2 shows the descriptive and statistical analysis for all three parameters extracted from the non-linear model. Long-term exploratory breadth most likely decreased (higher <*q*_*stat*_>) when playing under *restricted* space compared to *semi* (large effect) and *free* space (large effect) conditions. Also, a possible decrease was identified by comparing *semi* and *free* space scenarios (small effect). When playing under *restricted* space conditions, players’ exploratory dynamics quickly attained the stationary value (higher α) and likely decreased during the other two training situations. So, the larger the slope (α exponent), the quicker the players’ exploration. There were unclear differences between *semi* and *free* space conditions. Finally, the time lag in which the exploratory behaviour became saturated most likely (moderate/large effect) increased in the tasks under *semi* and *free* space conditions, respectively, compared to the *restricted* condition.

### Ball flow dynamics

**Fig 3:**
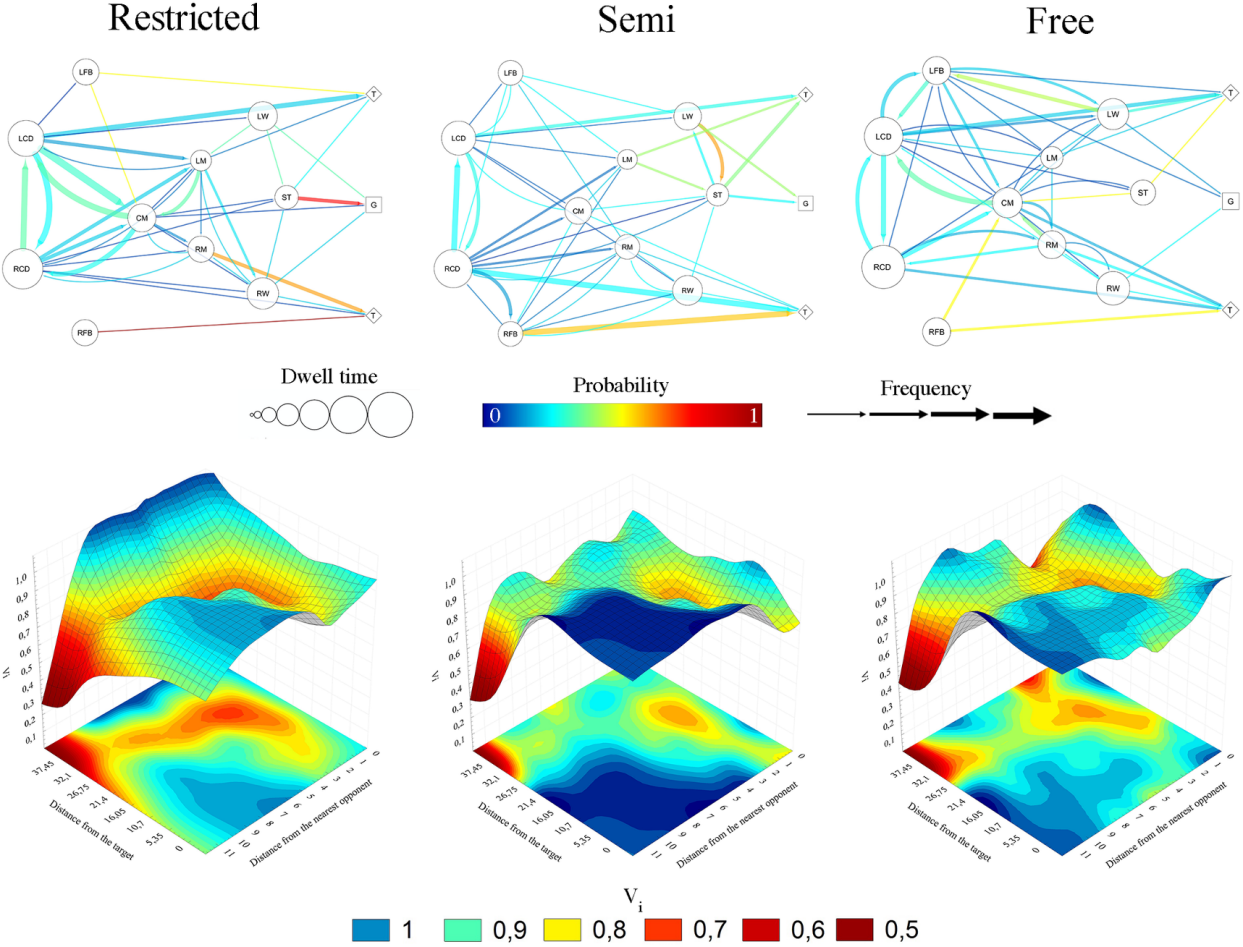
Upper panels: Network diagrams obtained from each task constraint. Size of nodes represents the mean time of players in possession of the ball. The width represents the frequency number of passes. Probability of passing interactions was depicted as the following soften scale: 0 –blue, 0.5 –yellow, 1 –red. Lower panels: Potential landscapes formed by two state (coordinative) variables of the player in possession of the ball (distance from the target and nearest opponent) under the three different task constraints. The 3D deeper wells correspond to 2D-projected more stable (i.e., more probable) red areas. The blue areas correspond to unstable coordinative states. Less stable coordinative states are more likely to decay into more stable states.

Table 2 shows the results of ball flow dynamics. The mean time (in seconds) during which players were in possession of the ball (Δ*t* ball possession) possibly decreased under the *free*-*space* condition (~-9%) compared to the *semi* space condition, while an unclear effect was shown between the *restricted* and the other two task constraints. Fig 3 shows that the mean time of players in ball possession was larger under restricted condition, specially central defenders.

**Table 2:** Descriptive analysis (mean±SD) of mean time of players’ ball possession, frequencies of passing interaction and frequencies of the player in possession of the ball’s configurations. Difference in means, uncertainty in the true differences, based on probability chances, and Standardized Cohen’s *d* differences among training game situations.

	Restricted	Semi	Free		Difference in means, %; ±90% CL	Chances for smaller/ similar/ greater value	Uncertainty in the true differences	Standardized Cohen’s d; ± 90% CL
				a)	-4.6 ±17.3	11/52/36	unclear	-0.11±0.41
Δt ball possession	3.19±1.47	3.03±1.08	2.71±0.73	b)	-7.1 ±21.0	12/41/47	unclear	-0.17±0.51
n	20	20	20	c)	-8.7 ±13.8	3/44/53	possibly ↓	-0.21±0.35
				a)	-15.6 ±24.2	5/30/65	unclear	-0.32±0.52
Passing interactions	0.72±1.27	0.57±0.94	0.72±1.01	b)	-1.3 ±20.4	17/61/22	unclear	-0.02±0.38
n	110	110	110	c)	25.1 ±26.4	83/17/1	likely ↑	0.42±0.39
				a)	-26.8 ±13.4	0/9/91	likely ↓	-0.37±0.22
Configuration space	3.22±6.18	2.63±4.26	2.40±3.08	b)	-34.9 ±9.9	0/0/100	most likely ↓	-0.51±0.18
n	108	108	108	c)	-4.6 ±15.9	2/86/12	likely trivial	-0.06±0.20

Note: Δ*t* = mean time; CL = confidence limits; ↑ = increase; ↓ = decrease. Comparisons between the three different training situations are identified as: a) *restricted* vs *semi*, b) *restricted* vs *free* and c) *semi* vs *free*

The Chi-square test did not show any significant differences (*p*<0.05) for each pair comparison. The passing interactions likely increased under the *free* space condition compared to the *semi* space condition (difference in means, %; ±90% CL: -25.1; ±26.4). Unclear differences were shown when comparing the *restricted* space condition with the other two task constraints, although a small effect (-0.32±0.52) was found between *restricted* and *semi* space conditions (see Table 2). As shown in the network for the *restricted* space situation (see upper panels of Fig 3), the most probable passing channel was found among players situated in the central corridor, connecting with the wings to score a goal or to pass to the forward for scoring. Not allowing the players to move out of their zone decreased the possibilities of passing interaction between full-backs and their teammates. This tendency changed when players were allowed to move to the adjacent sub-area, as did the probability of interaction between midfielders and forwards. In contrast, the probability of interaction with forwards decreased in the situation where players’ movements were not restricted. In addition, the probability of a player passing to the central midfielder increased.

### Player in possession of the ball’s configuration space

The Chi-square test shows significant differences in the frequency values when comparing *restricted* and *semi* space conditions [χ2 (107) = 369.96; *P*<0.001], *restricted* and *free* space conditions [χ2 (107) = 348.05; *P*<0.001] and *semi* and *free* space conditions [χ2 (107) = 222.30; *P*<0.001; η2 = 0.16]. Inferential magnitude-based analyses are presented in Table 2 (*restricted* vs *semi* space *conditions*, *restricted* vs *free* space conditions and *semi* vs *free* space conditions, respectively). The results showed a moderate negative effect when playing in the *restricted* space scenario (likely 26.8±13.4% and most likely 34.9±9.9% decrease compared to *semi* and *free* space conditions). When comparing *semi* and *free* space conditions, there were likely trivial (-4.6±15.9%) differences with a trivial effect (Table 2).

The high-value surfaces of the potential landscape define unstable coordinative states while the minimum values define highly stable coordinative states (see lower panels of Fig 3). The first thing to notice is that the changing task constraints brought about not only quantitative changes but also qualitative changes, i.e. bifurcations, in the configuration space. The stability profile and also the number of stable minima changed as a consequence of varying the task constraints. However, under all three task constraints, there were also invariant features of the potential landscape. On the one hand, a “free player” coordinative state was constantly unstable. This meant that the player in possession of the ball was five meters away from his nearest opponent. Under the *restricted* space condition, deeper valleys were depicted, showing that the player in possession of the ball worked within highly stable contexts. *Semi* space conditions, such as those where the ball was taken into distant zones, afforded less stable local states. However, when the ball was fewer than 30 meters from the scoring zone, the opponent delayed the ball carrier’s action (i.e., distance from the nearest opponent less than five meters), depicting other stable configurations for all three conditions. Moreover, all potential landscapes showed how the pressure increased when the players took the ball closer to the target zone.

## Discussion

This study aimed to present the influence of spatial restrictions on players’ movements on the exploratory dynamics of tactical behaviour, ball flow dynamics and performance contexts when players were in possession of the ball. The main finding suggests that restricting players’ movements out of their home sub-area enhanced their exploration in a short timescale, but not their long-term exploratory behaviour. Furthermore, results did not reveal that spatial restrictions help players to possess the ball in advantageous conditions, i.e. far from the opponent in advanced zones. However, allowing players to move out of their home sub-area fostered the ball flow dynamics and the inter-player passing relations.

In terms of exploratory dynamics, players produced a higher long-term exploratory breadth (i.e., lower <*q*_*stat*_>) when allowed to move out of their specific locations. While the results might seem obvious, previous studies have shown that increasing the space of interaction leads to decreases in the variability of players’ spatial distribution [42,59]. These differences may be due to the multivariate analysis used on here, which involved tactical actions, as well as to trajectories, distances, and movement-speed state variables. The players’ exploration quickly became saturated under restricted spatial condition. This means that players were constantly exploring new task solutions but quickly expired the whole set of possibilities for action. On the contrary, relaxing spatial restrictions increased long-term exploratory behaviour. Some previous results showed that a high number of opponents generally impaired long-term tactical exploration [48]. However, these constraints increased the probability of exploring certain specific coordinative states, e.g., depth mobility back to the defence.

The manipulation of task constraints changes the probabilities of emergent tactical behaviours and the exploration in different regions of the task solution space [55,60] Players’ behaviour can be defined by several variables that shows the relations between the system components. In this sense, performance coordinative variables, such as distance to opponents and the target can serve as parsimonious macroscopic descriptors of what happens at the microscopic level [53,61].

The depiction of these state variables on a performance configuration space gives a clear picture, thus allowing it to be determined whether or not the task constraints analysed foster an advantageous performance game scenario. For example, the *restricted* space condition allowed players to play the ball far from their nearest opponent when decreasing the distance from the target zone. This task constraint forced players to keep a large team surface area, thereby fostering penetration actions or passing the ball to a free teammate [7,49]. During the *semi* condition, this coordinative state had a greater degree of instability. Conversely, allowing players to move to the adjacent sub-area increased the stability of coordinative states, with the player in possession of the ball delayed in zones far from the target. The depth of the potential of the player in possession of the ball clearly showed how forbidding players to move out of their home sub-area can limit the exploration of task solutions yet foster the stability of concrete task solution states [48,62]. Moreover, it was shown that the change of task constraints changes not only the quantitative aspects of the game, e.g. modifying the stability degree of extant stable coordinative states, but also causes a formation of new stable states. This means that changes of task constraints may create qualitatively new contexts of exploration for players that cannot be interchangeable with respect to expected effects.

Network analysis allowed quantifying players performance [20] and identifying different player solicitations. In that sense, the right fullbacks were no able to succeed during their interventions with the ball in all three scenarios. However, playing under the *semi* space condition reduced the barriers to interacting with full-backs compared to the other two conditions. Previous network analysis in football has shown preferred passing channels and how they changed depending on the opposing team [25,63]. Positional constrains might be considered to simulate opponents’ defensive strategies. Allowing players to move on the adjacent sub-areas, wing opponents were able to press players located in the central corridor, leaving the fullbacks and wing forward players free of a marker, fostering the association with them. However, that task constraints lead to a lower centralized passing interaction patterns and higher intra-team well-connected passing relations, these behaviours are related with a better team performance [18,19]. Grund [64] found that high levels of interaction (i.e., passes per minute weighted by possession) led to increased team performance (i.e., goals). The more the players were in possession of the ball, the fewer passes per minute could be performed. According to this statement, relaxing players’ positional constraints would enable an increase in team performance regarding the quickness of ball flow dynamics and decreasing the players’ dwell time in possession of the ball. Larger passes samples would probably allow to identify the common features and emergent properties of the dynamic network topology under the effects of different task constraints [23,24].

In light of these results, when the coach used sub-spaces to constrain players’ behaviours, the exploratory behaviours of players quickly attained the stationary value, showing larger values of overlapped behaviours on a shorter timescale. Nevertheless, these positional restrictions helped to find advantageous scenarios for the player in possession of the ball. It has been demonstrated that stability in the degree of free movement of players can be constrained by changing the relative space per player by increasing the number of players [52]. Five-a-side formats increase the percentage of time spent from four to six meters away from the nearest opponent but decrease the probability of taking action from six meters free of opponents, as more advantageous contexts of play. Interestingly, Silva and colleagues [59] established that, with a larger (152 m^2^) relative space per player, the percentage of time spent in numerical advantage very likely increased by changing the field dimension instead of the number of players. Under imbalanced small-sided game conditions, the radius of free movement most likely increased for those players who competed in numerical superiority [47]. From these and previous results, a clear role of the constraints with respect to coaches’ goals arises. If the goal of the coach is to generally increase the individual player’s exploratory behaviour, he/she would have to allow players to move to other sub-areas of the pitch for the training task. If the goal is to elicit and/or stabilize specific coordinative states, then restricting positions to a concrete zone may be an optimal training context. Therefore, coaches should carefully design their training tasks. For this purpose, reading later studies that evidence the tactical effects of modifying tasks constraints is advisable. In addition, more research is needed in order to better understand the effects of task constraints involving large numbers of players, in which the spatiotemporal dynamics of game constraints is emphasized.

Previous research using a football match highlighted the importance to create local imbalance on specific zones of the area of play [43]. Further research should include other relational variables, such as angles, in order to analyse the relation between at least three system components (e.g. ball-player-target). Moreover, multivariate analysis (e.g. Principal Component Analysis) would allow knowing the (co) relation between the already relational variables captured characterizing the dynamical structure of tactical behaviour under the influence of task and environmental constraints. The dynamic nature of constraints and their evolution on different time scales should be also considered for investigation.

## Conclusion

Performance analysis can be improved by providing information about the dynamics of football games. The combination of ball events and positional data can help coaches to understand the effect of a task constraint on individual and collective behaviour. Moreover, the combination of relational variables can give a clear picture about the tactical performance of players and its dynamics. Network analysis is widely used during football matches, but its use in training settings is still limited. Here, it has been reported new knowledge on how ball flow dynamics could be constrained through pitch positioning restrictions. Spatial restrictions did not stimulate the long-term players’ exploratory breadth, but increased the rate of exploration to perform different tactical solutions on a shorter timescale. Dynamic overlap might be considered as a potential order parameter for performance analysis in sport. The depiction of relational variables also provide relevant information for tactical performance in a macroscopic level. Overall, coaches can foster players’ exploration and/or to stabilize concrete coordinative states by constraining the players’ space of interaction behaviour. In this sense, depending on the training goals of the coaches i.e. enhancing the exploration of task solutions or focusing on smaller set of task solutions, they can manipulate the constraints to attain each of them.
